# Long Bones Exhibit Adaptive Responses to Chronic Low‐Dose‐Rate Ionizing Radiation despite its Lifespan‐Shortening and Carcinogenic Effects on C57BL/6 Mice

**DOI:** 10.1002/jbm4.10688

**Published:** 2022-12-03

**Authors:** Masaoki Kohzaki, Akira Ootsuyama, Toshiaki Abe, Manabu Tsukamoto, Toshiyuki Umata, Ryuji Okazaki

**Affiliations:** ^1^ Department of Radiobiology and Hygiene Management Institute of Industrial Ecological Sciences, University of Occupational and Environmental Health Kitakyushu Japan; ^2^ Department of Radiation Biology and Health School of Medicine, University of Occupational and Environmental Health Kitakyushu Japan; ^3^ Radioisotope Research Center Facility for Education and Research Support, University of Occupational and Environmental Health Kitakyushu Japan; ^4^ Department of Orthopedic Surgery, School of Medicine University of Occupational and Environmental Health Kitakyushu Japan

**Keywords:** ANIMAL MODELS, BIOCHEMICAL MARKERS OF BONE TURNOVER, BONE QCT/ΜCT, ORTHOPEDICS, RADIOLOGY

## Abstract

Ionizing radiation (IR) is a well‐known carcinogen. High‐dose‐rate (HDR) IR is known to damage long bones (in terms of mass and structure), but the relationships among dose rates (particularly low‐dose‐rate [LDR] IR), long‐bone condition, cancer incidence, and lifespan shortening remain elusive. The aim of this study was to elucidate the effects of LDR‐IR on long‐bone condition by comparing the long‐term consequences of HDR‐ and LDR‐IR exposure in mice. We utilized micro–computed tomography (μCT) scans of the long bones at similar ages (mean 77–96 weeks) to compare mice receiving approximately equivalent total doses of internal LDR‐IR or external HDR‐IR starting at 4 weeks of age, and their respective control groups. The lifespan‐shortening effects of LDR‐IR and HDR‐IR were similar in these mixed‐sex cohorts. Notably, compared to HDR‐IR mice, mice internally exposed to chronic LDR‐IR with continuous hypohematopoiesis showed a significantly higher trabecular bone connective density [femur: 247% (*p* = 0.0042), tibia: 169% (*p* = 0.0005)] and midshaft cortical bone thickness/area (femur: 130% [*p* = 0.0079]/120% [*p* = 0.021], tibia: 148% [*p* = 0.0004]/129% [*p* = 0.002]). Consistent with this result, when comparing 26–32 weeks post‐IR with 2–8 weeks post‐IR, compared to HDR‐IR‐treated mice, LDR‐IR‐treated mice exhibited higher levels of an osteoblast marker (OPG; *p* = 0.67 for HDR‐IR, *p* = 0.068 for LDR‐IR) and lower levels of an osteoclast marker (CTX‐I; *p* = 0.0079 for HDR‐IR, *p* = 0.72 for LDR‐IR) despite significantly higher levels of inflammatory markers (CCL2 and CXCL1; *p* = 0.36–0.8 for HDR‐IR, *p* = 0.013–0.041 for LDR‐IR). These results suggest that long bones under chronic LDR‐IR stress may exhibit an adaptive response to stresses such as chronic inflammation associated with IR‐induced lifespan shortening. © 2022 The Authors. *JBMR Plus* published by Wiley Periodicals LLC on behalf of American Society for Bone and Mineral Research.

## Graphical abstract

The relationships among low‐dose‐rate (LDR) ionizing radiation (IR), long‐bone condition, cancer incidence, and lifespan shortening remain elusive. Thus, we performed micro–computed tomography scans of the long bones in high‐dose‐rate (HDR)‐ and LDR‐IR‐irradiated mice at comparable ages

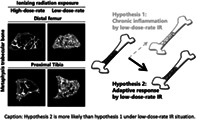

## Introduction

Although ionizing radiation (IR) is frequently used in cancer treatments, it is also considered a potent carcinogen^(^ ^1^ ^)^ and induces dose‐dependent deleterious effects in mammals. Several mouse models have been established to demonstrate the effect of IR on carcinogenesis over a long latency period.^(^ ^2^ ^)^ One of these mouse models using C57BL/6J mice is constructed by total‐body X‐ray irradiation with four weekly doses of 1.75 Gy starting at 8 weeks of age; this dosage induces thymic lymphomas in over 90% of mice.^(^ ^3^ ^)^ In contrast, low‐dose‐rate (LDR; 1.2 mGy/h) IR does not induce thymic lymphomas.^(^ ^4^ ^)^ Another model utilizes internal deposition (20 μGy/min) of the low‐energy β particle‐emitter calcium‐45 (Ca‐45; effective half‐life: 163 days); this exposure shortens the lifespan of mice in a dose‐dependent manner and induces cancer at very high (> 20 Gy) doses.^(^ ^5^ ^)^ Although both LDR and high‐dose‐rate (HDR) IR can induce cancer in mice, LDR‐IR may have conflicting effects on the risk of cancer depending on the genetic background of the mouse strain.^(^ ^2^, ^6^ ^)^

In humans, the biological effect of chronic exposure to LDR radiation is well studied with data from the Techa River Cohort (TRC) in Russia which includes nearly 30,000 residents of the Techa River area.^(^ ^7^ ^)^ For a long period, Techa River residents experienced γ‐ray exposure and internal exposure to mainly Sr‐89, Sr‐90‐, and Cs‐137 via their food, water, and milk; these dietary components were contaminated by waste produced by the Mayak Radiochemical Plant that was dumped in the river between 1949 and 1956. The residents of the Techa River region who were exposed to 0.7–1.0 mGy/year and whose cumulative dose exceeded 2–3 Gy developed symptoms of chronic radiation syndrome (CRS), including suppressed immunity and hematopoiesis.^(^ ^8^ ^)^ After following the TRC over 50 years, the cumulative bone marrow dose was calculated to be 66 mGy/year on average, and these individuals exhibited an excess relative risk for leukemia in a dose‐dependent manner.^(^ ^7^, ^8^ ^)^ These results suggest that protracted LDR‐IR exposure can induce long‐term carcinogenic effects, similar to those induced by HDR‐IR exposure in individuals from Hiroshima and Nagasaki who survived nuclear attack.^(^ ^9^ ^)^ Therefore, the total dose of IR is an important determinant of subsequent human health.^(^ ^10^ ^)^ Importantly, the rates of cortical bone resorption as well as the doses absorbed by the bone marrow and bone surface were significantly reduced in Techa River residents.^(^ ^11^ ^)^ However, no study has investigated the mass and structure of long bone (i.e., the femur and tibia) of Techa River residents using direct three‐dimensional (3D) methods.

**Fig. 1:**
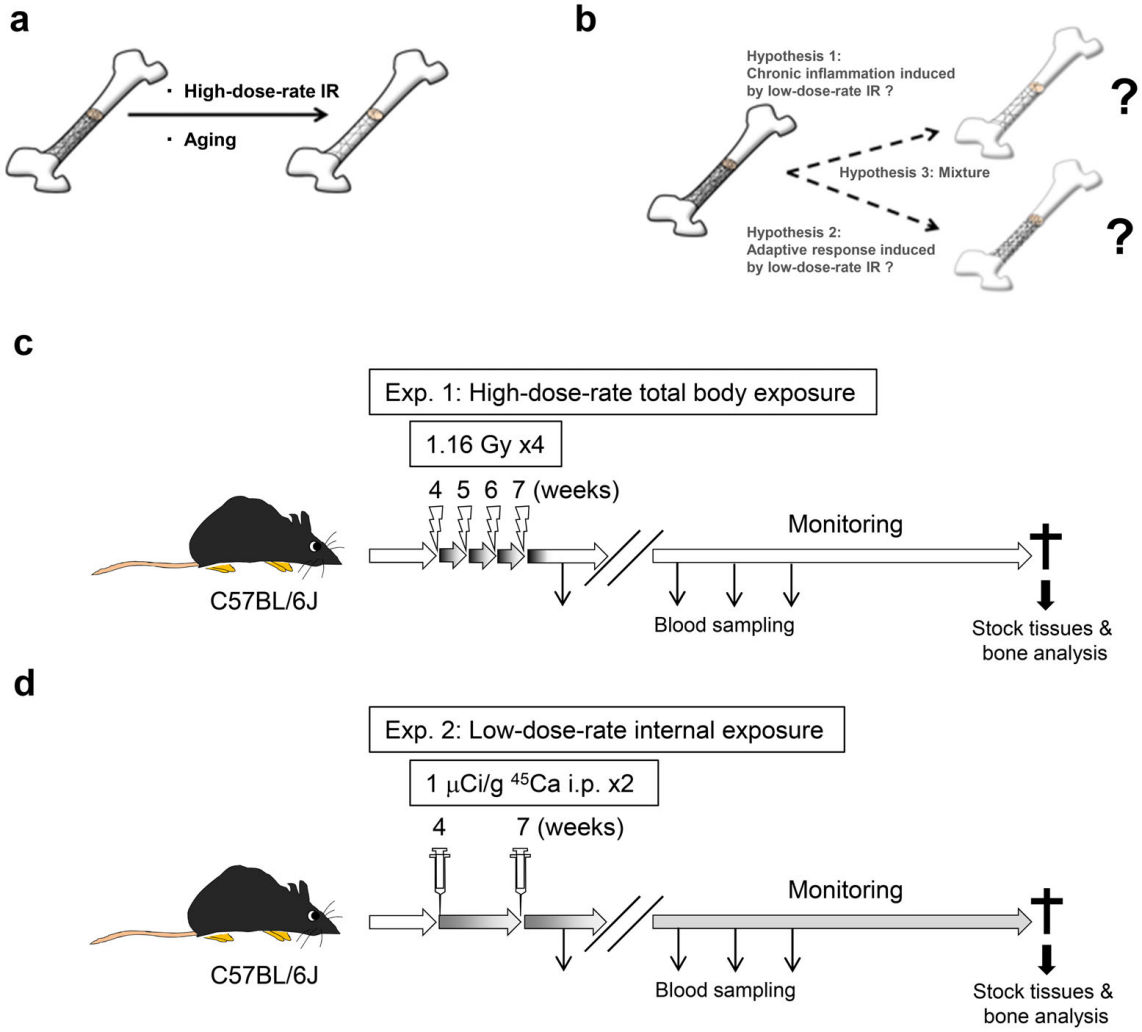
Schematic overview of the two independent in vivo experimental procedures. (*A*) Image of a partial cross section of the femur. The lines represent the bone connectivity density. It is widely accepted that bone mass and structure decrease with age or upon application of HDR‐ IR. (*B*) LDR‐IR induces chronic inflammation and an adaptive response. Chronic inflammation may decrease bone condition (hypothesis 1); alternatively the adaptive response induced by LDR‐IR may inhibit this decrease in bone condition (hypothesis 2). Hypothesis 3 represents a combination of the other two hypotheses. (*C*) In the external HDR‐IR‐exposure experiment, 4‐week‐old C57BL/6J mice were subjected to weekly X‐ray exposure (1.16 Gy) and were irradiated four times for a total of 4.64 Gy. (*D*) In the internal LDR‐IR‐exposure experiment, 1 μCi/g Ca‐45 was injected into 4‐ and 7‐week‐old C57BL/6J mice. The estimated doses absorbed by bone and marrow were calculated (see Methods). To determine the numbers of white and red blood cells and serum biochemical properties, peripheral blood samples were collected over time. When the mice died, carcasses and tissues were fixed and preserved in formalin for later histological and bone analyses.

Bone marrow, which is found in the central cavity of long bones, is a radiation‐hypersensitive organ that contains heterologous cells, including hematopoietic stem cells (HSCs), mesenchymal stromal cells, and endothelial cells.^(^ ^12^ ^)^ Numerous molecular‐level studies have revealed that microenvironments, including that of the endosteal osteoblastic and perivascular bone marrow niche, can regulate HSCs directly or indirectly.^(^ ^12^ ^)^ Dysregulation of the hematopoietic progenitors or stem cell niche may contribute to carcinogenesis.^(^ ^13^ ^)^ At the bone tissue level, it is well known that either aging or HDR‐IR can damage bone mass and structure (Fig. 1*A*).^(^ ^14^, ^15^ ^)^ However, whether LDR‐IR damages bone mass and structure or prevents bone‐health decline remains unknown. LDR‐IR, environmental chemicals, and infection can cause chronic inflammation,^(^ ^16^ ^)^ which is associated with age and can induce osteoporosis,^(^ ^17^ ^)^ characterized by an increased risk of fracture and low bone mass; hence, LDR‐IR may cause low bone mass (Fig. 1*B*; hypothesis 1). In contrast, LDR‐IR can also induce an adaptive response.^(^ ^2^, ^4^ ^)^ Bone is a flexible organ that can respond adaptively to stress, such as mechanical loading,^(^ ^18^ ^)^ and LDR‐IR may stimulate an adaptive response that reduces the decline in bone mass and structure associated with aging (Fig. 1*B*; hypothesis 2). Because the rates of cortical bone resorption were significantly reduced in internally exposed Techa River residents and C57BL/6 mice are useful models for reflecting age‐related bone condition in humans,^(^ ^11^, ^14^, ^18^ ^)^ hypothesis 2 seems likely, although there is no direct evidence to date. Bone turnover markers of osteoblastogenesis (procollagen type I N‐terminal propeptide, PINP; osteoprotegerin, OPG) and osteoclastogenesis (C‐terminal cross‐linked telopeptides of type I collagen, CTX‐I) in serum are well established in mice and humans.^(^ ^19^ ^)^ In addition, inflammatory markers, including the C‐C motif chemokine 2 (CCL2) cytokine and C‐X‐C motif chemokine ligand 1 (CXCL1) chemokine, have been identified in mouse and human sera.^(^ ^20^ ^)^ Therefore, bone turnover and inflammation can be monitored over time. To date, the relationships among internal LDR‐IR, HDR‐IR, long‐bone mass and structure at the tissue level, and cancer incidence have not been fully elucidated. Therefore, in this study, we systematically compared total‐body HDR‐IR, internal exposure to LDR‐IR, cancer incidence, and lifespan parameters in C57BL/6J mice.

## Materials and Methods

### Mouse experiments

C57BL/6J mice were purchased from SLC (Shizuoka, Japan). Mice were housed in cages containing four to six animals each for external exposure to X‐ray irradiation and in cages containing one to two animals each for internal exposure to Ca‐45 under pathogen‐free conditions on a 12‐hour dark/light cycle at an ambient temperature of 20–26°C. The mice had ad libitum access to a sterile diet (MF, Oriental Yeast Co., Japan). We allocated the mice into groups in a blinded manner, and all animal experiments were performed in accordance with guidelines issued by Animal Research: Reporting of In Vivo Experiments (ARRIVE), the Laboratory Animal Research Center, and the Institutional Animal Care and Use Committee of the University of Occupational and Environmental Health, Japan. The Ca‐45 experiments were performed in accordance with Japanese law on the prevention of radiation hazards due to radioisotopes.

### Thymic lymphoma induction via external exposure

Four‐week‐old mice were not irradiated (10 males, eight females) or irradiated weekly (nine males, 11 females) at a dose of 1.16 Gy (150 kV; 20 mA; filter: 0.2 mm Cu and 0.5 mm Al; MBR‐1520R‐3; Hitachi Power Solutions, Japan) in a pie cage divided into 12 rooms, four times in total (Fig. 1*C*).^(^ ^3^ ^)^ For dosimetry assessments of mouse external exposure, we inserted an ionization chamber‐type dosimeter (Semiflex 31013 ionization chamber, PTW‐Freiburg, Germany) into mouse carcasses and calculated the exposure dose in the dorsal and abdominal regions. Mouse peripheral blood was collected over time from the submandibular vein by a 0.5‐mm Goldenrod Animal Lancet (MEDIpoint, New York, USA) to evaluate the recovery of the white blood cell (WBC) count and red blood cell (RBC) count after IR. The body weights of the mice were regularly measured. Moribund mice were euthanized via cervical dislocation and necropsied. Biopsies and organ tissues were fixed with formalin and subjected to histological and micro‐positron emission tomography (PET) analyses. During the experiment, mice occasionally died for unknown reasons; for these mice, we could not accurately perform a necropsy.

### Internal exposure

#### Experimental procedure

Ca‐45 (calcium‐45, ^45^Ca) was purchased from the Japan Radioisotope Association (NEZ013, PerkinElmer, Waltham, MA, USA). A total of 1 μCi/g (740 kBq for a 20‐g mouse) Ca‐45, which was diluted with 0.9% saline (200 μl), or control 0.9% saline (200 μl) was injected intraperitoneally (i.p.) into mice twice at 4 weeks old and 7 weeks old (four males, four females). The mice were provided a standard pellet diet (MF, Oriental Yeast Co., Japan). A 20‐g mouse consumes approximately 3 g/day (32.1 mg calcium/day) of the standard pellet diet (Fig. 1*D*). Hence, the Ca‐45 content was much lower than the daily calcium intake because 1,480 kBq Ca‐45 contains 4 μg calcium. The noninjected control mice were housed under the same environmental conditions as the mice injected with Ca‐45 (four males, five females). Mouse peripheral blood was collected over time from the submandibular vein by a 0.5‐mm Goldenrod Animal Lancet to monitor the WBC and RBC counts. The body weights of the mice were regularly measured, and moribund mice were euthanized via cervical dislocation and necropsied. The mouse carcasses and organ tissues were immersed in 10% neutral‐buffered formalin and preserved whole for dissection to assess the effects of LDR‐IR by histological and micro‐PET analyses. Occasionally, mice died for unknown reasons and an accurate necropsy could not be performed.

#### Calculation of the internal exposure dose

**Fig. 2:**
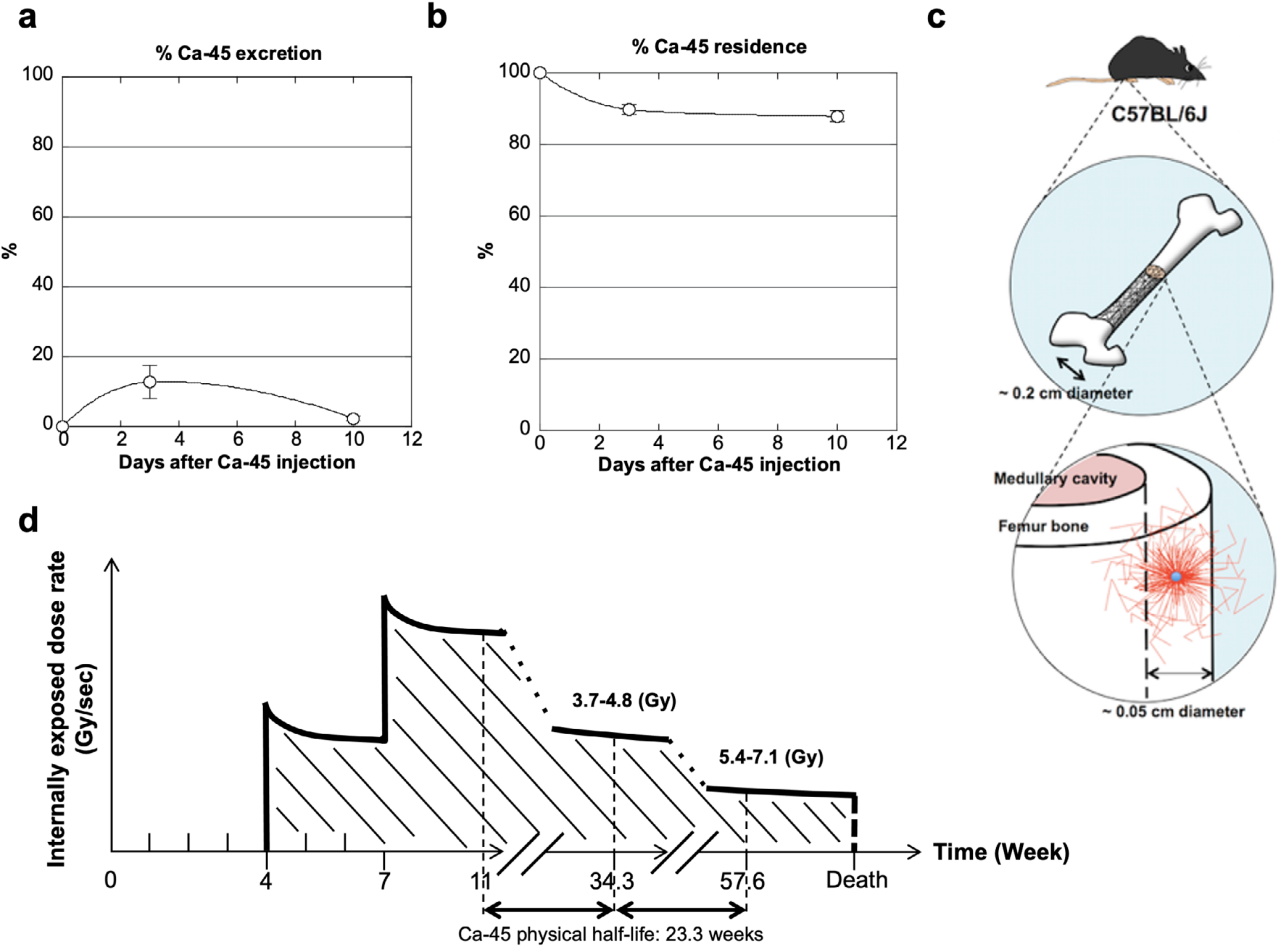
Dosimetry calculation for C57BL/6 mice internally exposed to Ca‐45 with an injection. (*A*) Percentage of Ca‐45 excreted after the injection is shown. Mean values of 12.9% (SD: 4.7%) on day 3 and 2.3% (SD: 0.7%) on day 10 were obtained. Only a small percentage of Ca‐45 was excreted 10 days after injection. (*B*) The percentage of Ca‐45 remaining after the injection is shown. Mean values of 89.7% (SD: 1.4%) at day 3 and 87.9% (SD: 1.6%) at day 10 were obtained. The nearly identical residual percentages of Ca‐45 at 3 days and 10 days after injection suggest that Ca‐45 was mainly present in the bone within a few days. (*C*) Images of bone internally exposed to Ca‐45 (small blue circle). Most low‐energy β particles remained within the bone, although some minor β particles may have eluded bone incorporation (red lines). (*D*) Schematic overview of accumulated Ca‐45 in the internal exposure condition after two injections. The diagonal lines under the bold line indicate the cumulative internal exposure dose. The diagonal lines slanted in the opposite direction indicate the time of omission. The physical half‐life of Ca‐45 is 23.3 weeks, and the estimated absorbed doses in long bones and marrow at 34.3 weeks and 57.6 weeks of age are indicated as examples (see Methods). Note that the presented numbers are estimates of radioactive calcium in the mineralized bone matrix.

Mice were reared in cages with a stainless‐steel wire mesh inserted 2 cm above the bottom of the cage to allow mouse feces to fall to the bottom and thus prevent excrement from sticking to the mice. Although Ca‐45 is excreted mainly in urine,^(^ ^21^ ^)^ we collected all excrement, including feces and urine, by pulverizing the feces and adding 400 mL of water over the stainless‐steel wire mesh. One day later, the solution was mixed well, and 1 mL of the solution was analyzed using a liquid scintillation counter (TriCarb 2900TR, PerkinElmer, Waltham, MA, USA). Observation of three mice at each time point was used to calculate the mean (SD) excretion and residual rates of Ca‐45 (Fig. 2*A*, *B*). The biological half‐life of Ca‐45, which accumulates in bones, is 49 years, and our excretion and residual rates were consistent with this value. Therefore, we used the physical half‐life of Ca‐45 (163 days) to calculate the internal exposure dose. The dose (Gy) of Ca‐45 absorbed by tissue was determined based on the reported calculation.^(^ ^22^ ^)^ Trabecular bone is considered a solid mineral material. The mean energy of β emissions by Ca‐45 is 0.077 MeV (Emax = 0.257 MeV); we also calculated the equilibrium dose constant of Ca‐45, which ranged from 1.23–1.60 × 10^−14^ Gy∙kg/Bq∙s. Berger's point kernels determined by the Monte Carlo method, which indicate the absorbed dose distributions for β point sources in water/tissue, were used to determine the absorbed fractions of β emissions in bone.^(^ ^23^ ^)^ The range of β particles from Ca‐45 in water/tissue is 0.062 cm, and some β‐particle energy from Ca‐45 can elude bone incorporation in mice (Fig. 2*C*). The range of β particles from Sr‐89 is 0.84 cm in water/tissue, and the fraction absorbed by mouse bone is 0.4.^(^ ^22^ ^)^ The fraction of Ca‐45 absorbed by mouse bone is approximately 0.9, which is nearly twice that of Sr‐89.^(^ ^24^ ^)^ In contrast, the dose‐ and dose‐rate‐effectiveness factor (DDREF) values for LDR‐IR are approximately half those of HDR‐IR.^(^ ^10^ ^)^ Therefore, we did not further correct the estimated doses of Ca‐45 absorbed by mouse bone when comparing the lifespan‐shortening and carcinogenic effects of HDR‐IR and LDR‐IR in the mouse experiments. The cumulative absorbed doses in mouse femurs, tibias, and marrow at 34.3 weeks, 57.6 weeks, and 80.9 weeks were in the following ranges: 3.7–4.8 Gy, 5.4–7.1 Gy, and 6.3–8.2 Gy, respectively (Fig. 2*D*).

### Histological analysis

Mouse tissues fixed with formalin were dehydrated and embedded in paraffin by conventional methods. Then, 5‐μm sections were cut from formalin‐fixed paraffin‐embedded blocks and transferred onto glass slides. The sections were stained with hematoxylin and eosin (H&E) using standard procedures. Images were obtained by a BZ‐X700 instrument (Keyence, Osaka, Japan).

### Microstructural analysis of trabecular and cortical bone

Formalin‐soaked mouse carcasses or tissues were washed with water and scanned with a μCT system (CosmoScan GX; Rigaku, Tokyo, Japan). The age‐matched unexposed and noninjected control mice were either assessed live under anesthetization with sevoflurane (193‐17791, Wako, Japan) or as carcasses. The trabecular bones of the distal femoral and proximal tibial metaphysis were analyzed by μCT at a resolution of 10 × 10 × 10 μm^3^ (90 kVp, 88 μA, 533.33 milliseconds of integration time). A median‐type filter with a 5 × 5 × 5 kernel was utilized. We analyzed the metaphyseal volume at a height of 1.0 μm, commencing 0.5 μm proximal to the growth plate of the proximal tibia and distal femur.^(^ ^25^ ^)^ Cortical bone was assessed from 50 midshaft slices from the femur and tibia. The minimum threshold for bone density was 334 mg/cm^3^, determined through correlations with phantoms of known density. The bone microstructure parameters were evaluated using Analyze 12.0 software (AnalyzeDirect, KS, USA) based on the following guidelines^(^ ^26^ ^)^: BV/TV (%), Tb.Th (mm), trabecular separation (Tb.Sp; mm), trabecular number (Tb.N; 1/mm), structure model index (SMI), connectivity density (Conn.D; 1/mm^3^), total cross sectional area inside the periosteal envelope (Tt.Ar; mm^2^), cortical bone area (Ct.Ar; mm^2^), cortical area fraction (Ct.Ar/Tt.Ar; %), and average cortical thickness (Ct.Th; mm). We analyzed bone mass and structure in a blinded manner.

### ELISA

Peripheral blood (50–500 μL) from irradiated and nonirradiated C57BL/6 mice was regularly collected every several weeks with 1.5 mL microcentrifuge tubes from the submandibular vein with a 0.5‐mm Goldenrod Animal Lancet (MEDIpoint, New York, USA). After the blood was collected, the hemorrhagic area was compressed with KimWipes to stop the bleeding. The collected blood was allowed to clot for at least 30 minutes at room temperature. The tubes were centrifuged at 3000*g* for 10 minutes at 4°C. Serum was collected and stored at −80°C until use. All measurements were performed with an ELISA kit or Duoset ELISA kit in accordance with the manufactures' instructions. The serum levels of the bone resorption marker CTX‐I were measured by ELISA (AC‐06F1, IDS, RatLaps Collagen I). The serum levels of the bone formation markers PINP and OPG were measured by ELISA (AC‐33F1, IDS) and DuoSet ELISA (DY459, R&D systems), respectively. The serum levels of the CCL2 cytokine and CXCL1 chemokine were measured by DuoSet ELISA (DY479 for CCL2, DY453 for CXCL1, R&D Systems). A standard curve was generated for each protein, and concentrations were extrapolated from the standard curve.

### Statistical analysis

All statistical tests were two‐sided. A trend toward significance was set at a *p* value <0.075, statistical significance was indicated by a *p* value < 0.05. For two‐group comparisons, the data were subjected to the Kolmogorov–Smirnov test to evaluate the normality of distributions between the two groups. If the *p* value was less than 0.05, the data were considered to have a nonnormal distribution. Normally distributed samples were analyzed using Welch's *t* test. Samples with a nonnormal distribution were analyzed using the Mann–Whitney U test. *P* values from the Kaplan–Meier survival analysis were determined by the Gehan–Breslow–Wilcoxon test. For comparisons among four groups, the data were subjected to a one‐way ANOVA with Tukey's post hoc test. No statistical methods were used to predetermine the sample size. All analyses were performed using GraphPad Prism (version 6.0 g, La Jolla, CA).

## Results

### C57BL/6 mice subjected to roughly equivalent doses of HDR‐ and LDR‐IR had shorter lifespans than corresponding control mice

**Fig. 3:**
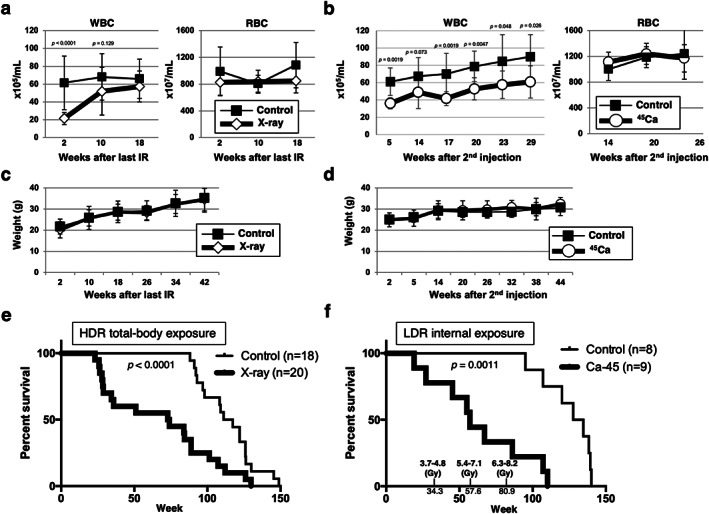
C57BL/6 mice administered similar total estimated doses of HDR‐IR and LDR‐IR had shortened lifespans compared with the controls. (*A*, *B*) The white blood cell (WBC) and red blood cell (RBC) counts in peripheral blood from control and X‐ray‐irradiated mice (*A*) as well as control and Ca‐45‐injected mice (*B*) are plotted as the mean (SD). The *p* values were determined by the Mann–Whitney U test or Welch's *t*‐test. (*C*, *D*) Body weight changes in mice treated (*C*) with or without HDR‐IR or (*D*) with or without LDR‐IR are plotted as the average weights (SDs). (*E*, *F*) Kaplan–Meier survival analysis for each method of irradiation. The total number of mice in each group is indicated in parentheses. The *p* values (*p* = 0.0011, *p* < 0.0001) indicated on the plots were determined by the Gehan–Breslow–Wilcoxon test. (*E*) In the external HDR‐IR‐exposure experiment, 10 males and eight females were allocated to the control group, whereas nine male and 11 female mice were allocated to the exposure groups. (*F*) In the internal LDR‐IR‐exposure experiment, four male and four female mice were in the control group, whereas five male and four female mice were included in the Ca‐45 injection groups. The estimated absorbed doses in bone and marrow are indicated (see Methods).

The radiation dose rate can affect an individual's cancer risk in a dose‐dependent manner; the total dose of IR is associated with cancer risk and lifespan regardless of fractionation.^(^ ^10^ ^)^ However, long‐term internal exposure to LDR‐IR has received less attention than external exposure to HDR‐IR due to the complexity of the calculations (Figs. 1 and 2, see Methods). We first generated a well‐established thymic lymphoma model in C57BL/6 mice for external HDR‐IR experiments (Fig. 1*C*). The WBC count was used to assess the recovery of lymphocytes after IR. As expected, the WBC count was significantly decreased at 2 weeks after HDR‐IR and recovered at 10 weeks after IR, while the RBC count did not change (Fig. 3*A*).

**Fig. 4:**
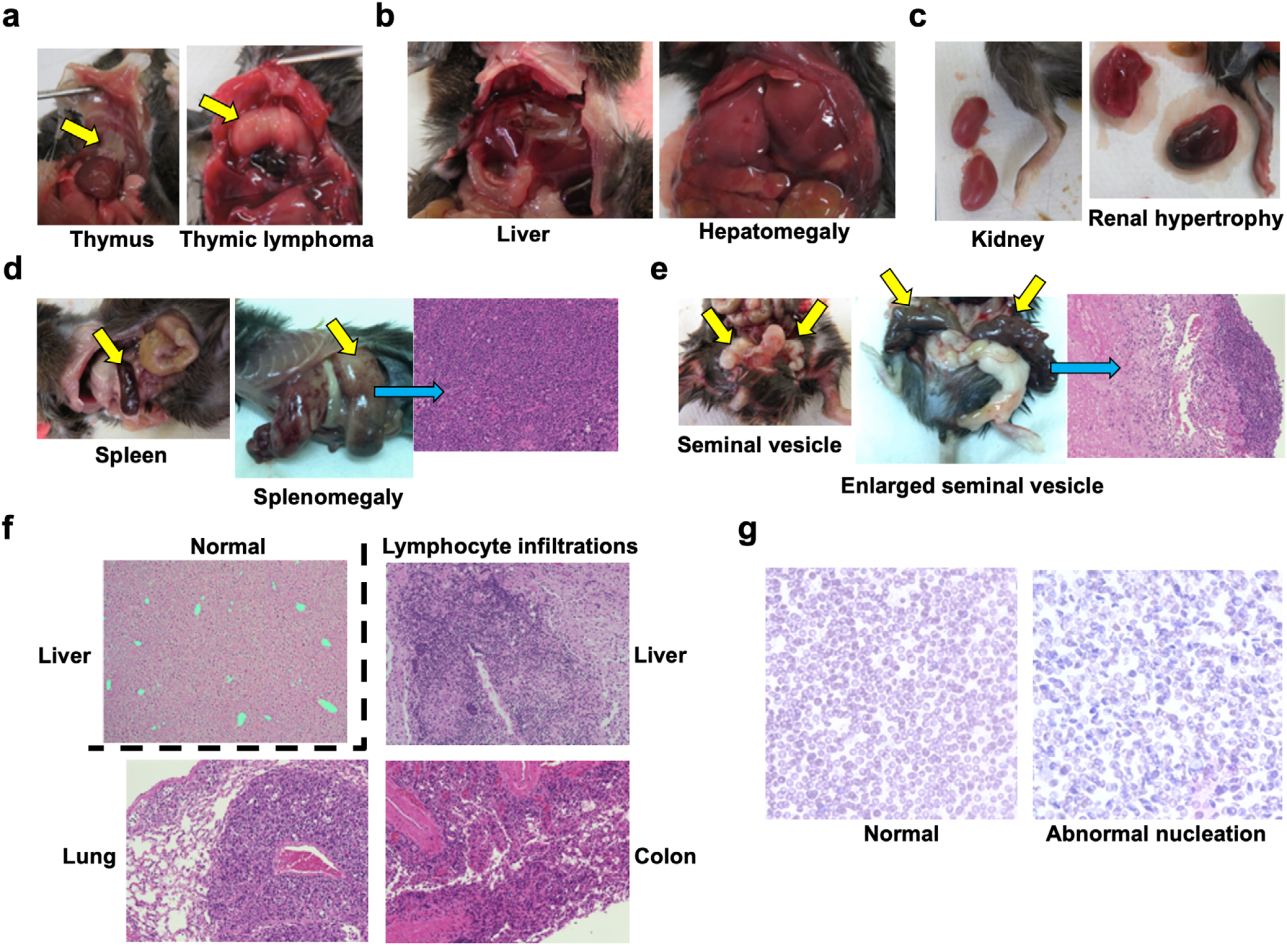
Representative images of tissues and histological analysis of C57BL/6 mice. (*A*–*E*) Normal images of tissues and representative images of abnormal tissues. (*A*) Representative images of thymus and thymic lymphoma (yellow arrow). (*B*) Representative images of liver and hepatomegaly. Other tissues are also shown for size comparison. (*C*) Representative images of kidney and renal hypertrophy. The mouse leg is also shown for size comparison. (*D*) Representative images of spleen (left, yellow arrow) and splenomegaly (middle, yellow arrow). Splenic tissue was filled with lymphocytes (right). H&E staining; magnification ×20. (*E*) Representative images of seminal vesicles (left, two yellow arrows) and enlarged seminal vesicles (middle, two yellow arrows). Some part of the enlarged seminal vesicle was filled with concentrated lymphocytes (right). H&E staining; magnification ×20. (*F*) Representative histological images of normal liver (upper left) as well as lymphocyte infiltration in the liver (upper right), lung (lower left), and colon (lower right). Typical perivascular infiltration in lung tissue. H & E staining; magnification ×20. (*G*), Representative images of normal (left) and abnormal (right) smears. Abnormal nucleated red blood was stained blue with Giemsa. Magnification ×20.

To administer a similar total dose of internal LDR‐IR, we next conducted low‐energy β‐particle Ca‐45 injection experiments (Figs 1*D* and 2, see Methods). Mice injected with Ca‐45 were chronically exposed to the β emitter Ca‐45 at a dose of 5 to 29 mGy/day, which meets the criteria for a LDR exposure (< 144 mGy/day).^(^ ^2^ ^)^ In stark contrast to the HDR‐IR group, in the LDR‐IR group, the WBC count was consistently lower in the mice injected with Ca‐45 than in the control mice for up to 29 weeks after injection (Fig. 3*B*). However, the body weights and RBC counts of HDR‐IR‐ and LDR‐IR‐treated mice did not differ from those of the corresponding controls (Fig. 3*A–D*). In addition, both experiments revealed that the irradiated mice had significantly shorter lifespans than the control mice (Fig. 3 *E*, *F*). HDR‐IR mainly induced thymic lymphoma from 28 to 35 weeks of age (Figs. 3*E* and 4 *A*, Table 1).^(^ ^3^ ^)^ Consistent with a reported study, LDR‐IR did not induce thymic lymphoma in C57BL/6J mice (Table 1).^(^ ^4^ ^)^ We observed splenomegaly, hematoma, and senescence‐associated phenotypes, including enlarged seminal vesicles, in older control and IR‐treated mice (Fig. 4, Table 1).^(^ ^27^ ^)^ In particular, we observed a significant increase in the number of infiltrating lymphocytes in X‐ray‐irradiated mice at later time points (Fig. 4 *F*, Table 1), supporting a relatively high incidence of lymphoma in the C57BL/6 mouse strain.^(^ ^28^ ^)^ Long‐lived control mice had higher percentages of bowel obstructions or enlarged seminal vesicles than IR‐treated mice (Table 1), suggesting that the IR‐treated mice might have died from carcinogenesis accelerated by IR rather than the physiological decline associated with aging. In summary, aside from HDR‐IR‐induced thymic lymphoma, the application of approximately equivalent total doses of internal LDR‐IR and external HDR‐IR resulted in similar lifespan‐shortening consequences.

**Table 1:** Biopsy Results in HDR‐IR‐ and LDR‐IR‐Exposed C57BL/6J mice^a^

	HDR‐IR (X‐ray)		LDR‐IR (Ca‐45)	
	Control (*n* = 18)	X‐ray (*n* = 20)	Control (*n* = 8)	Ca‐45 (*n* = 9)
Thymic lymphoma	0	7 (35%)	0	0
Lymphoma^b^	2 (11.1%)	2 (10%)	1 (12.5%)	2 (22.2%)
Splenomegaly	4 (22.2%)	2 (10%)	1 (12.5%)	1 (11.1%)
Lymphocytic infiltrate^c^	1 (5.6%)	9 (45%)	N/A	N/A
Ascites	0	1 (5%)	0	1 (11.1%)
Cardiac hypertrophy	0	0	0	1 (11.1%)
Enucleation	0	0	0	1 (11.1%)
Hepatomegaly	1 (5.6%)	1 (5%)	0	0
Renal hypertrophy	0	1 (5%)	0	0
Hemoperitoneum	2 (11.1%)	1 (5%)	0	1 (11.1%)
Hematoma	1 (5.6%)	2 (10%)	1 (12.5%)	1 (11.1%)
Pleural effusion	0	1 (5%)	0	0
Bowel obstruction	2 (11.1%)	0	0	0
Vertebral disc degenerative disorder	0	0	0	1 (11.1%)
Osteosarcoma	0	0	0	1 (11.1%)
Hepatobiliary neoplasm	2 (11.1%)	1 (5%)	0	0
Renal tumor	0	1 (5%)	0	0
Enlarged seminal vesicles^d^	6 (60%)	2 (22.2%)	2 (50%)	2 (40%)
Unknown^e^	7 (39%)	6 (30%)	4 (50%)	3 (33.3%)

^a^ One diagnosis of each type was scored when a single animal had two or more diagnoses that were of different types. However, some lesions may have been missed by biopsy because we investigated obviously abnormal lesions.

^b^ Not otherwise specified.

^c^ Infiltrated lymphocytes were observed in lung, colon, and liver tissue samples. Note: Histological analysis of the Ca‐45 group was difficult and is thus presented as not available (N/A).

^d^ Observed in male mice; the percentage was calculated only among males. Ten of 18 male mice from the HDR‐IR control group, nine of 20 male mice from the HDR‐IR group, four of eight male mice from the LDR‐IR control group, and four of nine male mice from the LDR‐IR group were used for this analysis.

^e^ Mice without a diagnosis due to unknown/undeterminable cause of death. The cause of death is thus listed as unknown.

### Long‐bone mass and structure in LDR‐exposed mice were maintained with aging compared to those in HDR‐exposed mice

**Fig. 5:**
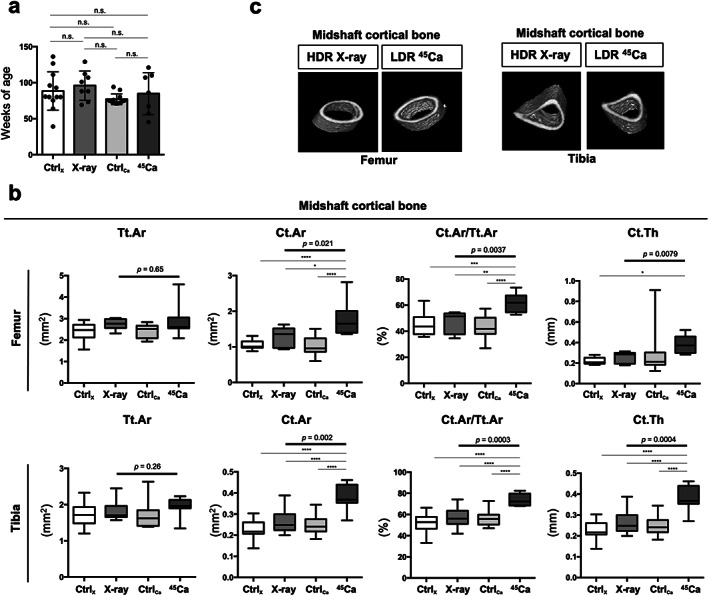
Comparison of midshaft cortical mass and structure among mice treated with whole‐body HDR‐IR (external exposure), those injected with LDR‐IR (internal exposure), and their controls at similar ages. (*A*) Comparison of age (in weeks) at which microstructural parameters of the cortical and trabecular bones of HDR‐IR control (Ctrl_X_), HDR‐IR (X‐ray), LDR‐ IR control (Ctrl_Ca_), and LDR‐IR (Ca‐45) groups were assessed. The graph shows the mean (SD), and the data were analyzed with a two‐sided one‐way ANOVA with Tukey's post hoc test. n.s., not significant. (*B*) Comparison of midshaft cortical microstructural parameters of femur (*n* = 12 for Ctrl_X_, *n* = 11 for X‐ray, *n* = 11 for Ctrl_Ca_, *n* = 9 for Ca‐45) and tibia (*n* = 11–13 for Ctrl_X_, *n* = 10 for X‐ray, *n* = 17 for Ctrl_Ca_, *n* = 13 for Ca‐45). Tt.Ar, total area; Ct.Ar, cortical bone area; Ct.Ar/Tt.Ar, cortical area fraction; Ct.Th, average cortical thickness. The data are depicted by the box‐and‐whisker plot showing the medians, first and third quartiles (boxes), and overall ranges (whiskers) and were subjected to a two‐sided one‐way ANOVA with Tukey's post hoc test to determine *p* values (*p < 0.05, **p < 0.01, ***p < 0.001, ****p < 0.0001). (*C*) 3D images of midshaft cortical area of femurs (left two images) and tibias (right two images) of HDR‐IR (X‐ray‐exposed) mice (left side) and LDR‐IR (Ca‐45‐injected) mice (right side). Representative images of mice at 80 weeks old in X‐ray group and mice at 79 weeks of age in the Ca‐45 group are shown.

The bone mass and structure of humans and animals, including mice, decline over time.^(^ ^14^ ^)^ In addition, HDR‐IR is known to accelerate bone loss^(^ ^15^, ^29^ ^)^; in contrast, the mechanism by which LDR‐IR affects bone mass and structure remains unclear. Therefore, we analyzed the microstructures of trabecular and cortical bone of mice at similar ages in four different groups (Fig. 5*A*). Although the total areas of the midshaft cortical femur and tibia were similar among groups (Fig. 5*B*, left), in the LDR‐IR group, the midshaft cortical areas of the femur and tibia were significantly higher than those in the other groups (Fig. 5*B*, middle left). As a result, the quotient of the cortical area and the total area (Ct.Ar/Tr.Ar) in the midshaft cortical area of the femur and tibia in LDR‐IR mice were significantly higher than those in the other groups (Fig. 5*B*, middle right). In addition, the average cortical thickness (Ct.Th) in the midshaft femur in LDR‐IR mice tended to be higher than that of the other groups, and the Ct.Th values of the midshaft cortical tibia were significantly higher in the LDR‐IR group than those in the other groups (Fig. 5*B*, right).

**Fig. 6:**
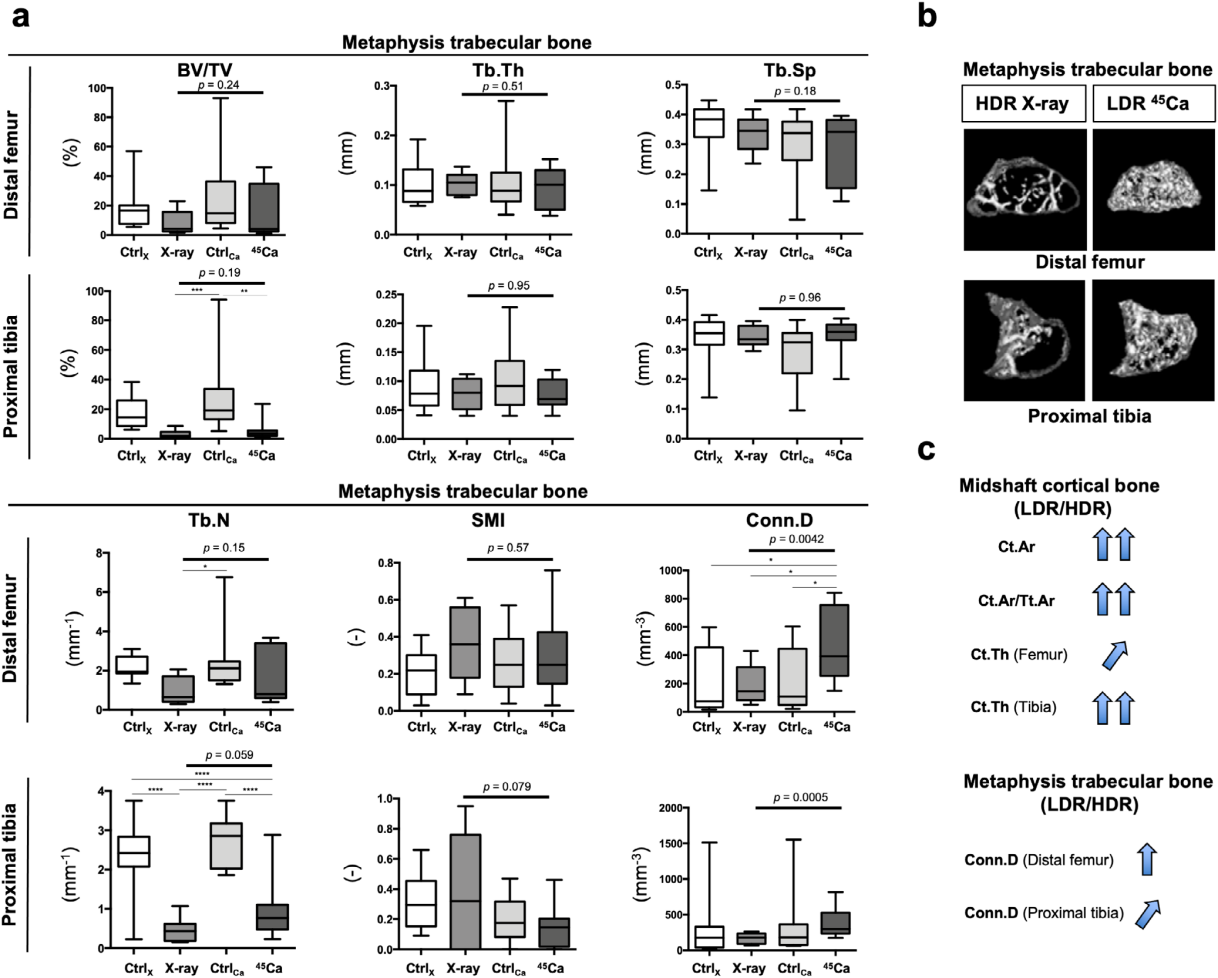
Comparison of trabecular metaphysis mass and structure among mice treated with whole‐body HDR‐IR (external exposure), those injected with LDR‐IR (internal exposure), and their controls at similar ages. (*A*) Comparison of microstructural parameters of femoral trabecular metaphysis (*n* = 12–13 for Ctrl_X_, *n* = 9 for X‐ray, *n* = 17 for Ctrl_Ca_, *n* = 12 for Ca‐45) and tibia (*n* = 13–14 for Ctrl_X_, *n* = 11 for X‐ray, *n* = 13–18 for Ctrl_Ca_, *n* = 12 for Ca‐45). BV/TV, bone volume; Tb.Th, trabecular thickness; Tb.Sp, trabecular separation; Tb.N, trabecular number; SMI, structure model index; and Conn.D, connectivity density. The data are depicted by the box‐and‐whisker plot showing the medians, first and third quartiles (boxes), and overall ranges (whiskers) and were subjected to a two‐sided one‐way ANOVA with Tukey's post hoc test to determine *p* values (*p < 0.05, **p < 0.01, ***p < 0.001, ****p < 0.0001). (*B*) 3D images of metaphysis trabecular bones of the distal femurs (upper) and proximal tibias (lower) of HDR‐IR (X‐ray‐irradiated) mice (left side) and LDR‐IR (Ca‐45‐injected) mice (right side). Representative images of mice at 73 weeks old in the X‐ray group and mice at 110–121 weeks of age in the Ca‐45 group are shown. (*C*) Summary of significant differences or different trends of trabecular and cortical bone microstructures between HDR‐ and LDR‐IR mice.

Indeed, we observed that the midshaft cortical bone mass and structure of Ca‐45‐injected mice were maintained compared to those of X‐ray‐irradiated mice (Fig. 5*C*). Next, to confirm the phenotypes in the LDR‐IR group, we analyzed the microstructures of the trabecular bone. The Conn.D of trabecular bone in Ca‐45‐injected mice was significantly maintained compared to that of X‐ray‐irradiated mice, particularly the distal femur (Fig. 6*A*). Again, we observed that the metaphysis trabecular bone mass and structure of Ca‐45‐injected mice were maintained compared to those of X‐ray‐irradiated mice (Fig. 6*B*). Overall, compared to the long bones of mice subjected to HDR‐IR, those of mice subjected to chronic LDR‐IR were maintained despite the lifespan‐shortening and carcinogenic effects of LDR‐IR on C57BL/6J mice (Fig. 6*C*).

### LDR‐IR‐exposed mice tended toward osteoblastogenesis rather than osteoclastogenesis and exhibited increased inflammation over time compared with HDR‐IR‐exposed mice

**Fig. 7:**
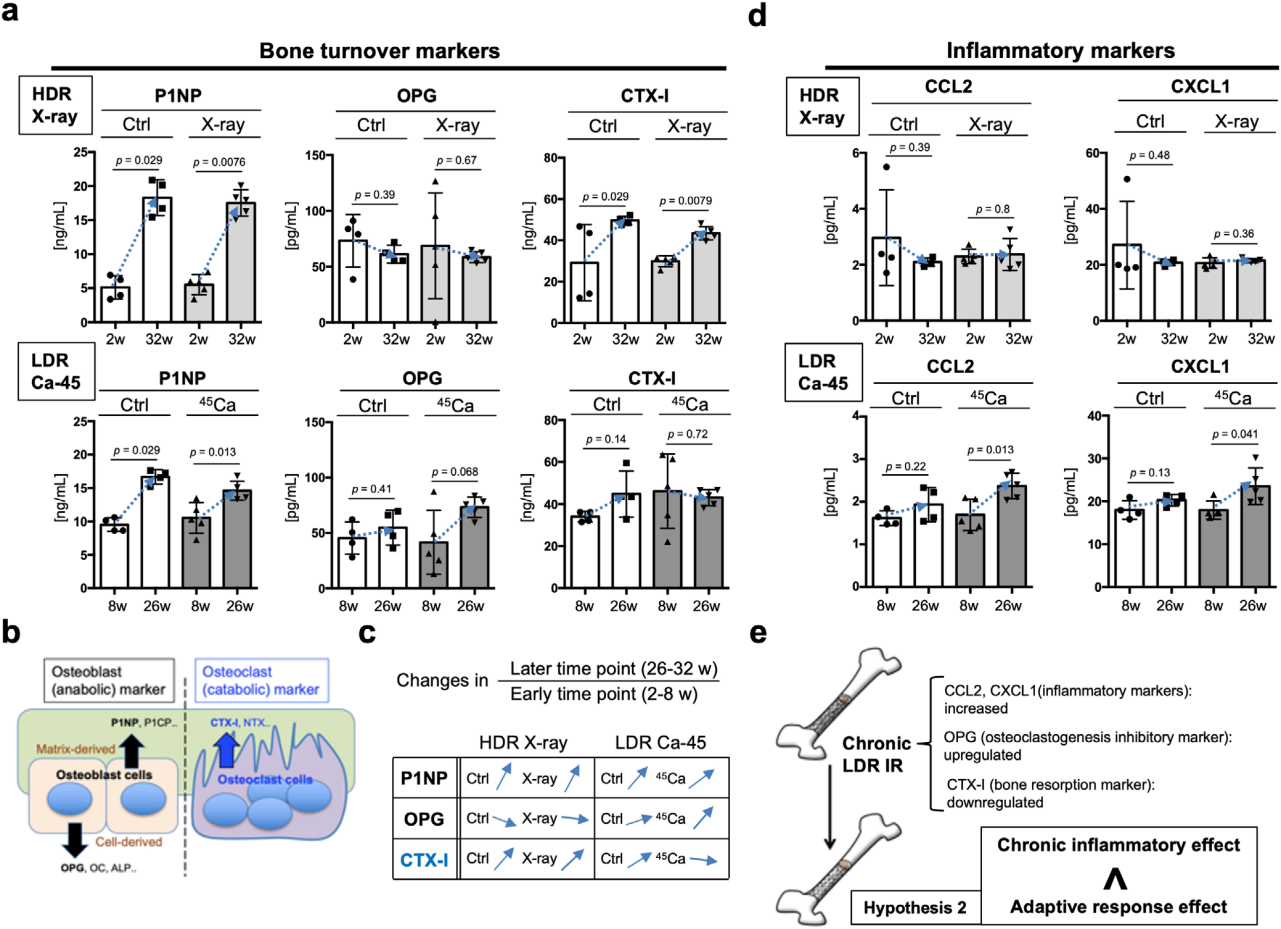
Comparison of levels of bone turnover markers and inflammatory markers in C57BL/6 mice exposed to whole‐body HDR‐IR (external exposure) or injected with LDR‐IR (internal exposure). (*A*) The biochemical levels of bone turnover markers of osteoblastogenesis (P1NP, left; OPG, middle) and osteoclastogenesis (CTX‐I, right) in control mice (two males, two females) and mice exposed to whole‐body HDR‐IR (three males, two females) at 2 weeks and 32 weeks are shown in the upper lane. Their levels in control mice (two males, two females) and mice internally exposed to LDR‐IR (three males, two females) at 8 weeks and 26 weeks of age are shown in the lower lane. (*B*) Schematic summary of bone markers used in this study. Bone markers are produced by two different pathways of origin, cell‐derived factors and matrix‐derived factors. (*C*) To easily compare changes over time (i.e., calculate the ratio of later time points (26–32 weeks) to early time points (2–8 weeks)), the trends over time (arrow) of all of the examined groups are shown. (*D*) The biochemical levels of inflammatory markers, CCL2 (left) and CXCL1 (right), in the control mice (two males, two females) and mice subjected to whole‐body HDR‐IR (three males, two females) at 2 weeks and 32 weeks of age are shown in the upper lane. Their levels in control mice (two males, two females) and mice internally exposed to LDR‐IR (three males, two females) at 8 weeks and 26 weeks of age are shown in the lower lane. (*E*) Osteoblastogenesis markers dominated osteoclastogenesis markers in mice chronically exposed to LDR‐ IR compared to mice externally exposed to HDR‐IR mice, with a small but significant increase in inflammation over time. Thus, this study supports hypothesis 2 since the adaptive response of long bones to chronic LDR‐IR was predominantly observed rather than a chronic inflammatory effect induced by chronic LDR‐IR.

Next, we analyzed the bone turnover markers at early time points (2–8 weeks after IR) and later time points (26–32 weeks after IR) to gain insight into the mechanisms underlying the differential effects of HDR‐IR and LDR‐IR on long bones over time (Fig. 7*A*). P1NP and OPG served as bone formation markers, and CTX‐I served as a bone resorption marker (Fig. 7*B*).^(^ ^19^ ^)^ The expression of P1NP constantly increased over time in all of the examined groups (left, Fig. 7*A*). In contrast, OPG showed a declining trend over time in the HDR‐IR group (upper middle, Fig. 7*A*) and an increasing trend over time in the LDR‐IR group (lower middle, Fig. 7*A*). In particular, OPG was expressed at higher levels in mice injected with Ca‐45 than in control mice (lower middle, *p* = 0.41 in the Ctrl group and *p* = 0.068 in the Ca‐45 group, Fig. 7*A*). The expression of the bone absorption marker CTX‐I increased over time in all the examined groups except for mice injected with Ca‐45 (right, Fig. 7*A*), suggesting that bone resorption over time was suppressed in mice injected with Ca‐45. Taken together, these results demonstrate that the mice injected with Ca‐45 showed a tendency toward osteoblastogenesis, rather than osteoclastogenesis, over time (Fig. 7*C*).

Finally, mouse inflammation was assessed using two independent inflammation markers, CCL2^(^ ^30^ ^)^ and CXCL1,^(^ ^31^ ^)^ to confirm that chronic LDR‐IR induced inflammation over time. The levels of CCL2 and CXCL1 were not altered in the HDR‐IR group over time (upper, Fig. 7*D*), while their levels significantly increased in Ca‐45‐injected mice (lower, Fig. 7*D*). These data suggest that chronic LDR‐IR biased bone metabolism toward formation rather than resorption, despite inducing low levels of inflammation (Fig. 7*E*), supporting hypothesis 2 (Fig. 1*B*).

## Discussion

Due to the ease of manipulation and dose calculations, most studies have employed short‐term exposure to HDR X‐rays or γ‐rays to induce cancer in mice. In contrast, the effects of chronic internal LDR‐IR exposure to radioactive nuclides depend on nuclide deposition in the target organs, decay over time, and the identity of the radioactive nuclides. Mouse organs are relatively small compared with the range of β particles; thus, estimating the absorbed dose is complex (Fig. 2). Hence, LDR‐IR has received much less attention than HDR‐IR. However, understanding the effects of long‐term LDR‐IR is essential because many occupational workers, including those in the nuclear and radiation therapy industries, are exposed to LDR‐IR for long periods.^(^ ^32^, ^33^, ^34^ ^)^ When assessing a very low dose of radiation administered over a long period, determination of the total dose and dose rate is necessary to evaluate the effects of IR on individual mammals. In this study, we systematically compared LDR‐IR and HDR‐IR to clearly identify the LDR‐IR effects on long‐bone mass and structure at the tissue level.

Hematopoiesis was shown to occur after exposure to LDR‐IR, but the WBC count was consistently lower in mice injected with Ca‐45 than in the control mice for up to 29 weeks after the injection (Fig. 3*B*). This result suggested that the Ca‐45 incorporated into the long bones constantly emitted low‐energy β particles and that the accumulated dose of IR suppressed hematopoiesis. The cumulative absorbed dose around the long bones damaged some radiosensitive cells, which are important for the differentiation of WBCs among the heterologous cells in bone marrow, and resulted in insufficient delivery of functional clones into the peripheral blood.^(^ ^35^ ^)^ Therefore, the maintained effect of long‐term LDR‐IR on the long‐bone mass and structure might have been masked by the dominant effect of the accumulated LDR‐IR on lymphocyte differentiation, which is required to prevent carcinogenesis and lifespan shortening in C57BL/6J mice (Fig. 3*F*). The constant decrease in the WBC count of mice caused by long‐term LDR‐IR is similar to that found in the Techa River residents who presented with CRS symptoms and hematopoiesis suppression (Fig. 3*B*).^(^ ^8^, ^10^ ^)^ Indeed, the dose rate we administered to the mice (5.01–29 mGy/day) was more than 10‐fold higher than that shown to cause a twofold increase in the frequency of lymphopenia in the TRC (0.33 mGy/day). In addition, the mouse lifespan is much shorter than the human lifespan; if we had administered the human dose rate to the mice in the current study, we would presumably have observed no signs of lifespan shortening. Therefore, the mouse results obtained in this study can be partially translated to humans.

Both bone volume (BV/TV) and trabecular number (Tb.N) of the proximal tibia were significantly lower in irradiated mice compared to nonirradiated controls, regardless of the dose rate (Fig. 6A). Although the biological mechanism underlying the reduction in only these factors later in life in mice exposed to radiation as juveniles is unclear, these bone changes may serve as indicators of radiation exposure later in life. The trabecular bone connectivity density (Conn.D) contributes more to the biomechanical strength of bone than the trabecular thickness (Tb.Th) or bone mineral density.^(^ ^36^ ^)^ Hence, the mice treated with long‐term LDR‐IR exhibited a higher trabecular bone Conn.D at death and may have experienced less alteration in bone biomechanical strength than mice subjected to HDR‐IR treatment (Fig. 6C). In addition, Techa River residents exposed to chronic LDR‐IR had lower cortical bone resorption rates than nonexposed residents; these rates correlated with the dose absorbed by the bone.^(^ ^11^ ^)^ Consistent with this finding, chronic LDR‐IR biased mice toward bone formation rather than bone resorption (Fig. 7*A–C*). Interestingly, the inhibition of bone resorption is a therapeutic target to prevent trabecular bone loss and fractures associated with menopause.^(^ ^37^ ^)^ In addition, long‐term LDR‐IR may actively stimulate bone formation by recruiting osteoblasts through the osteocyte network toward the bone surface.^(^ ^38^ ^)^ Indeed, strontium ranelate, a bone anabolic agent that behaves like calcium, can inhibit bone resorption activity by osteoclasts and enhance osteoblast function to promote bone formation in mammals.^(^ ^39^ ^)^ Some studies have reported that endochondral ossification or osteoprogenitors are required for the maintenance or formation of HSC niches, which are indispensable for hematopoiesis.^(^ ^40^, ^41^ ^)^ Since we observed continuously decreased WBC counts (Fig. 3*B*) and a trend toward osteoblastogenesis over time in mice exposed to chronic LDR‐IR (Fig. 7*A–C*), long‐term internal LDR‐IR exposure (via Ca‐45) may have stimulated both ossification and HSCs. Such continuous stimulation in the hematopoietic system could restrict the lymphoid differentiation potential (Fig. 3*B*) and shorten individual lifespans due to tumorigenesis (Fig. 3*F*).^(^ ^13^ ^)^ The levels of OPG, but not P1NP, increased significantly over time in the LDR‐IR‐treated mice compared to the HDR‐IR‐treated mice (Fig. 7*A*). P1NP propeptides are produced by not only bone but also other tissues, such as the cornea, skin, tendons, and vessels,^(^ ^42^ ^)^ and P1NP is cleared by liver endothelial cells via a macrophage receptor.^(^ ^43^ ^)^ These liver endothelial cells may have been affected by inflammation or inflammation‐dependent cellular functions^(^ ^44^, ^45^, ^46^ ^)^ and caused similar outcomes in the HDR‐IR and LDR‐IR groups (left, Fig. 7*A*). The inflammatory markers CCL2 and CXCL1 are also related to tumorigenesis and metastasis.^(^ ^30^, ^31^ ^)^ Because inflammation and cancer are tightly correlated,^(^ ^47^ ^)^ the significant upregulation of CCL2 and CXCL1 expression in the Ca‐45‐injected mice at 26–32 weeks after IR compared with 2–8 weeks after IR might have partially contributed to tumorigenesis under the chronic LDR‐IR conditions.

Our study has several limitations. First, we did not assess bone quality or bone strength by bone histological and organic (collagen content and crosslinking) analyses due to radioisotopic regulations in Japan. Second, several unknown factors, including individual differences, may have influenced the values calculated in this study, so the results cannot be completely verified. Third, we used a minimum number of mice for ethical and legal reasons, thereby limiting the sample size of this study. Fourth, although a nonirradiated control group was established for both the HDR and LDR conditions and comparative analysis was performed, differences due to the differences in exposure routes (internal versus external) should be considered. Last, the environment of C57BL/6J mice (i.e., specific‐pathogen‐free environment) differs from that of humans, and the environment affects the immune system responses of mice.^(^ ^48^ ^)^ Moreover, the results we obtained may have been affected by the differences in the genetic backgrounds of the mice.^(^ ^6^ ^)^ Thus, further studies will be required to address these limitations.

Adverse effects of zero gravity on bone condition, such as decreased bone mass, are well known during long‐term space flight missions. Based on the recent Radiation Assessment Detector (RAD) measurements obtained by the Curiosity on the way to Mars, the galactic cosmic radiation (GCR) of this space flight mission produced an estimated total equivalent dose in the bone marrow ranging from 1.84 to 2.48 mSv/day for a round‐trip Mars surface mission.^(^ ^49^ ^)^ Although the dose rates in mice with internal exposure to LDR‐IR were roughly comparable to those of GCR, the bone outcomes of these mice indicated a cumulative total exposure that exceeded the space‐relevant radiation dose within the first 10–15 weeks of the experiment upon measurements 1–2 years later. Notably, the relevance of the impact of GCR exposure is limited. Moreover, LDR GCR consists of γ‐rays, high‐energy charged particles, protons, and neutrons; the risks of GCR exposure depend on solar activity and many uncertainties and are difficult to accurately predict. Therefore, the increased risks of cancer, lifespan‐shortening effects, and maintained long‐bone mass and structure resulting from LDR (5–29 mGy/day) IR observed in C57BL/6 mice should be carefully considered when assessing the risks and benefits of space travel to astronauts. We should also consider the balance between these risks and benefits when using IR for radiation therapy. The dose used in our internal exposure condition was much higher than the safe dose. In fact, chronic internal LDR‐IR exposure significantly shortened the mouse lifespan (Fig. 3*F*). Hence, we do not recommend that continuous LDR β‐emitting exposure within the dose range we used be implemented to treat osteoporosis. In contrast, low‐energy β‐emitting particles are used in cancer treatment, particularly for metastatic cancer in the nuclear medicine field.^(^ ^50^ ^)^ Determining the lowest dose that stimulates the bone adaptive response without the risk of lifespan shortening is important. In summary, our results suggest that decreases in the long‐bone mass and structure over time can be prevented by long‐term LDR‐IR; however, exposure to this radiation still results in lifespan‐shortening and carcinogenic effects (Fig. 7*E*). These results may provide practical data on the health of not only occupational workers who are exposed to long‐term LDR‐IR in the nuclear, space exploration, and radiation therapy industries but also both metastatic cancer and osteoporosis patients.

## Disclosures

None of the authors has any conflicts of interest to declare.

## Author Contributions

**Masaoki Kohzaki:** Conceptualization; data curation; formal analysis; funding acquisition; investigation; methodology; project administration; resources; software; supervision; validation; visualization; writing – original draft; writing – review and editing. **Akira Ootsuyama:** Methodology; project administration; resources; supervision; validation. **Toshiaki Abe:** Methodology; resources; validation. **Manabu Tsukamoto:** Methodology; project administration; supervision; validation. **Toshiyuki Umata:** Resources; supervision. **Ryuji Okazaki:** Project administration; resources; supervision; validation.

### Peer Review

The peer review history for this article is available at https://publons.com/publon/10.1002/jbm4.10688.
